# The Importance of a Thorough Mechanical Training

**Published:** 1889-11

**Authors:** David Hepburn


ARTICLE II.

### THE IMPORTANCE OF A THOROUGH MECHANICAL TRAINING.

BY DAVID HEPBURN, L. D. S. ENG.

[An Introductory Lecture in Dental Mechanics delivered at the Dental Hospital of London.]

" *Gentlemen:*—We are met here this evening to commence our course of lectures on Dental Mechanics, and it is almost needless for me to remind you that in a department of your work so essentially practical in its nature as this, it is impossible for me to bring before you all the details of the various processes which pertain to the construction of artificial dentures. I must rather address myself to you as gentlemen who are already acquainted with many of these details, learned I trust, during your apprenticeship, and endeavour to select for your consideration those points which are least likely to have come under your notice in the routine work of the dental laboratory.

" I must not, however, allow this opportunity to pass by without saying one word on the great importance of this branch of your work. Much has been done of late years towards the preservation of the natural organs of mastication, but notwithstanding the advances of conservative dental surgery, there is a wide field left open in the department of prosthetic dentistry. Indeed, when we look around and note the increasing deterioration of the teeth and the universally acknowledged importance of the possession of masticating power, and feel, as we cannot fail to do, that with the poor material we have to work upon, our conservative operations must, in a large majority of cases, in time fail, we are justified in concluding that the services of the dental posthetist will be as greatly in demand in the future as they ever have been in the past. It therefore behoves us to cultivate this branch of our professional work with all assiduity.

"To cultivate it efficiently and intelligently, there is but one way open, and that is by diligent and constant application at the 'bench.' Nothing short of a period of intimate everyday hand-to-hand association with the practical details of dental mechanics as they come before us in the workroom will avail anything towards the acquirement of that necessary amount of knowledge which can lighten the difficulties of our so called 'mechanical cases,' when we begin to deal with them in the operating room.

"Theory without practical knowledge in most departments of work is a dangerous thing, but I think this especially applies to dental mechanics which never can, from the varying nature of the oral structures, be an exact art, and the practitioner who has neglected practical work in the workroom and relies upon theoretical knowledge gained from books or upon his own fancied ingenuity, will be constantly creating difficulties for himself and his patients by endeavouring to carry out in practice ideas which appear theoretically good but which are practical impossibilities. Earnest attention to this special branch of your professional studies will aid you also in another way, for it will help you to a better performance of your surgical operations. The hand of the dental surgeon must undergo training in some form or another so that it may gain that power, flexibility, dexterity and delicacy of touch which are essential elements for the proper handling of the various instruments employed in oral operations. The attainment of manipulative skill with the dental surgeon is an absolute necessity, and this skill should not be attained at the expense of the patient, but should be acquired, at any rate to a certain extent, before the student begins to handle the living subject. What better preparation for this dexterity can we find than the work which is, or should be, involved in the three years' apprenticeship in mechanics required by the curriculum of the Royal College of Surgeons?

"I cannot too strongly impress upon you the importance of this hand-training, for whether it be in taking impressions of the mouth, in inserting dentures, or in any of the varied operations of dental surgery, it matters not, but of this you may rest arrured that a firm, yet delicate touch will tend more to establish confidence on the part of your patients than any amount of rhetorical argument.

"Further, in acquiring an intimate and practical knowledge of dental mechanics, you will, so to speak, be able to command your own workrooms. However skillful may be the assisiant (if you be in a position to employ one), it must be borne in mind that he alone who has modelled the mouth and has studied the oral conditions and idiosyncrasies of the patient is able to direct the construction of the denture.

"The study of the model alone will never suffice for this. It therefore behoves every dental student to master in a really practical manner all the details of the various methods of accurately constructing dentures, and what is quite as important, the modifications requisite in certain cases, so that in designing the work he may not demand impossibilities from those who have to execute it.

"There is little rule or routine in the science of dental mechanics, except in its broad principles. Every case differs and must be treated according to its merits. Practice alone therefore teaches us what is requisite when difficulties present themselves and how these difficulties may best be overcome. Each individual case must be undertaken in the artist spirit. It must be entered into heartily for the work's sake and carefully considered in all its bearings. In the mind's eye every step in construction must be followed out,. these weighed side by side with the oral conditions and temperament of the patient, and this can only be done with hope of the attainment of a satisfactory result by the student who has devoted himself conscientiously to the study of this special branch of his profession.

"One more word on this subject; you must remember that there may come a time for all of us when the strain of the more intricate dental operations becomes too exacting and we are glad to turn with relief to those cases which we call our 'mechanical' ones. Our patients have advanced in years with us, and despite our endeavours to *save* teeth the ravages of nature need to be supplemented by art. Let me emphatically assert that no relief from anxiety and strain will be experienced in the treatment of these cases by dental surgeons who have not attained proficiency in dental mechanics in the days of their youth, for it appears to me that just as in the mastery of a musical instrument, proficiency in this branch of dentistry must be acquired early in life."

After describing the anatomy of the finger tips and showing that the sense of "touch" could be developed and not destroyed by a not too prolonged course of manipulative work in the dental laboratory, the lecturer proceeded to explain those points in the anatomy of the maxillary bones, temporo-maxillary articulation, actions of the muscles of mastication and structures of the tongue and palate, which bore special reference to the subject in hand, and dwelt at some length on the sense of taste and the actions of the pillars of the fauces, pharyngeal muscles and soft palate during the act of deglutition. He next referred to the pathological conditions associated with morbid states of the mucous membrane and subjacent structures, and insisted upon the necessity of inducing a healthy condition before attempting to insert any mechanical appliance. The rugæ of the palate and their distribution were explained, and all those points such as the papilla corresponding with the anterior palatine fossa, the frænum of the lip and tongue, the eminence of the base of the malar process of the superior maxillary bone and various reflexions of mucous membrane which it was desirable to guard from pressure, were pointed out. The changes occurring from absorption after loss of teeth and alterations in the jaws through age were illustrated by diagrams. After referring to the study of facial expression and the various forms and colours of teeth associated with diverse types and temperaments, the lecture terminated with a description of those points which should be considered in the preparation of the mouth for artificial teeth and the time for insertion of temporary and permanent plates.—*London Dental Record.*

